# High nature value farming systems in Europe: A dataset encompassing the environmental impact assessment of farms and extensive ruminant food products

**DOI:** 10.1016/j.dib.2024.111164

**Published:** 2024-12-04

**Authors:** M. Torres-Miralles, P. Jeanneret, M. Lamminen, F. Joly, B. Dumont, H. Tuomisto, I. Herzon

**Affiliations:** a Department of Agricultural Sciences, Faculty of Agriculture and Forestry, University of Helsinki, Latokartanonkaari 5, 00014, Finland; b Helsinki Institute of Sustainability Science (HELSUS), University of Helsinki, Yliopistonkatu 3, 00100, Finland; c ETSI Agronomica, Alimentaria y de Biosistemas, CEIGRAM Universidad Politécnica de Madrid, Spain; d Agroscope, Reckenholzstrasse 191, 8046 Zürich, Switzerland; e University of Clermont Auvergne, INRAE, VetAgro Sup, UMR Herbivores, 63122 Saint-Genès-Champanelle, France; f Natural Resources Institute Finland, Latokartanonkaari 9, 00790 Helsinki, Finland

**Keywords:** Extensive farming systems, Semi-natural habitats, Biodiversity, Agriculture, Life cycle assessment

## Abstract

High Nature Value (HNV) farming systems occur in areas where the major land use is agriculture and are characterized by their significance in promoting biodiversity and ecosystem services due to their extensive land use. Despite their importance for ecological and socio-economic resilience of rural regions, these systems are often overlooked in Life Cycle Assessment (LCA) studies due to challenges in data compilation, especially from small local farms and because of the diversity of production. To address this gap, we established an international collaborative network across Europe, involving professionals directly engaged with farmers, farmer associations, and researchers to collect data on HNV farms employing a developed questionnaire examining inputs and outputs, farm structures, and herd characteristics. Our dataset includes 41 farms and covers five European countries—Spain, France, Greece, Estonia, and Finland—spanning three bioregions of Mediterranean, Atlantic, and Boreal. Data, anonymised and integrated into a matrix, focus on such environmental impact indicators as greenhouse gas emissions (GHGs), biodiversity, land and water use, and fossil resource scarcity. We applied LCA using analytical tools such as the European Carbon Calculator (Joint Research Centre of the European Commission), OpenLCA 10.4., and the SALCA-BD expert system. Additionally, we utilised the LCA inventory Agribalyse 3.0 database to estimate the environmental footprint of four pivotal HNV products: goat cheese, cow milk, lamb, and beef. The main outcome is a unique and novel dataset for HNV farming systems, addressing critical gaps in available information. Our primary objective is to facilitate further investigations, empowering other researchers to expand and enhance their understanding of the environmental impact associated with HNV farming systems, drawing attention to a potential role of HNV farming systems in transitioning towards a more sustainable food production and consumption.

Specifications Table

**Table:** 

Subject	Agricultural Sciences			
Specific subject area	Agroecology, ruminant production			
Data format	Raw and analysed			
Type of data	Table			
Data collection	We initiated an international collaborative network across Europe, involving professionals directly engaged with farmers, farmer associations and researchers. We collected data from 41 farms with extensive ruminant production typical for the respective regions, developed a data collection methodology, which included the development of a questionnaire involving a detailed examination of inputs and outputs, farm structures and herd characteristics in five European countries: Spain, France, Greece, Estonia and Finland across three bioregions of Mediterranean, Atlantic and Boreal. The questionnaire was based on the data entry inputs of the Carbon Calculator of the Joint Research Centre of the European Commission (Tuomisto et al., 2015). We adapted question types to ensure clarity and ease of understanding for the participating farmers. We also incorporated expert opinions in the development of the questions to enhance the accuracy of the data collection process. The questionnaire, in excel format, was filled by each farmer with continuous support through phone to facilitate the process, addressing inquiries with the intention to harmonize raw data inputs and minimise biases. We estimated some of the parameters such as semi-natural grass intake and other grass intake from existing literature.			
Data source location	Farm	Country	Region (NUTS2)	NUTS3
	HNV1	Finland	Etelä-Suomi	Päijät-Häme
	HNV2	Finland		Southwest Finland
	HNV3	Finland		Päijät-Häme
	HNV4	Finland		Southern Ostrobothnia
	HNV5	Finland		South Karelia
	HNV6	Finland		Northern Ostrobothnia
	HNV7	Finland		Uusimaa
	HNV8	Finland		Pirkanmaa
	HNV9	Finland		Lapland
	HNV10	Finland		Uusimaa
	HNV11	Finland		Uusimaa
	HNV13	Greece	Θεσσαλία	Καρδίτσας
	HNV14	Greece	Θεσσαλία	Καρδίτσας
	HNV15	Greece	Θεσσαλία	Καρδίτσας
	HNV16	Greece	Θεσσαλία	Καρδίτσας
	HNV17	Greece	Θεσσαλία	Καρδίτσας
	HNV18	Greece	Θεσσαλία	Καρδίτσας
	HNV19	Greece	Θεσσαλία	Καρδίτσας
	HNV20	Greece	Θεσσαλία	Καρδίτσας
	HNV21	France	Pays de la Loire	Maine et Loire
	HNV22	France	Pays de la Loire	Maine et Loire
	HNV23	France	Pays de la Loire	Maine et Loire
	HNV24	France	Pays de la Loire	Maine et Loire
	HNV25	France	Pays de la Loire	Maine et Loire
	HNV26	Spain	Castile and Leon	Zamora
	HNV27	Spain	Andalusia	Granada
	HNV28	Spain	Catalonia	Barcelona
	HNV29	Spain	Catalonia	Girona
	HNV30	Spain	Castile-La Mancha	Toledo
	HNV31	Spain	Basque community	Biscay
	HNV32	Spain	Extremadura	Caceres
	HNV33	Spain	Andalusia	Sevilla
	HNV34	Spain	Galicia	Lugo
	HNV38	Estonia	Lääne-Eesti	Hiiu maakond
	HNV39	Estonia	Lääne-Eesti	Länneranna
	HNV40	Estonia	Lääne-Eesti	Läänemaa
	HNV41	Estonia	Lääne-Eesti	Hiiu maakond
	HNV42	Estonia	Lääne-Eesti	Hiiu maakond
	HNV43	Estonia	Lääne-Eesti	Pärnumaa
	HNV44	Estonia		Pärnumaa
	HNV45	Estonia	Lääne-Eesti	Saarenmaa
Data accessibility	Repository name: Figshare. Data identification number: 10.6084/m9.figshare.24942348. Direct URL to data: https://figshare.com/s/bbb175cd31450f7b31e7. Instructions for accessing these data: -			
Related research article	M. Torres-Miralles, V. Kyttä, P. Jeanneret, M. Lamminen, P. Manzano, H.L. Tuomisto, I. Herzon, Applying Life Cycle Assessment to European High Nature Value farming systems: environmental impacts and biodiversity, Agricultural Systems. Agric Syst 220 (2024) 104,096. 10.1016/j.agsy.2024.104096			

## 1 Value of the Data

• The significance of this dataset lies in addressing a substantial gap in the scientific understanding of High Nature Value (HNV). farming systems, particularly those dedicated to extensive ruminant production while maintaining biodiversity in Europe, which have received limited research attention in food assessments and have been underrepresented in current databases [1,2].
• The dataset provides a comprehensive Life Cycle Assessment (LCA) of extensive ruminant farming systems that offers a nuanced understanding of the diverse HNV farming systems and practices by integrating common environmental parameters with biodiversity values that contributes positively to the overall environmental impacts, therefore, addressing the limitations of previous studies that predominantly focus on adverse environmental impacts limiting the advance of environmentally conscious agricultural practices and dietary choices.
• The dataset offers novel estimations for ruminant production in semi-natural grasslands. These grasslands, characterised by minimal tree cover and diverse vegetation, are crucial for maintaining farmland biodiversity and ecosystem services in Europe.
• The dataset offers adaptable estimations allowing for comparisons, modifications and implementations to diverse ecological contexts. The dataset is designed to inform further research on HNV farming systems. Conclusions drawn from the dataset can also serve for informed decision making.
• By integrating product level impact data for key ruminant derived products — beef, lamb, cow milk and goat cheese — the dataset serves as a proxy for HNV products as an initial attempt to address the potential challenges in the scalability and representativeness of meat source foods under extensive farming practices in low input farming systems.

## 2 Background Section

The primary motivation for compiling this dataset arose from the necessity to address the considerable paucity of quantitative data concerning low-input farming systems, particularly those characterised by extensive ruminant production. This focus aimed to emphasise the existing relationship between ruminant grazing and the maintenance of biodiversity [3]. Despite advances in theoretical and methodological frameworks, existing research on animal source foods has predominantly relied on broad estimates based on a wide variation of farming systems and intensity of production practices. The diverse and region-specific nature of High Nature Value farming systems has posed significant challenges to the collection of specific data. To facilitate a transition towards sustainable animal source food production within healthy dietary frameworks, it is crucial to acquire quantitative data on low input production systems that allows for differentiations and adds nuance to the current debate. This data paper covered the most common ruminant production in European HNV farmlands — sheep, goat and cattle. This paper is designed to support a previous published study [1] by providing detailed explanations of assumptions and methodologies to clarify the underlying calculations on farming practices and other relevant aspects of ruminant production such as feed intakes required to assess the final environmental impact of HNV farming systems. This was necessary due to the complexity of the assessment and the constructed estimates.

## 3 Data Description

The dataset is available in Excel format and comprises comprehensive farm descriptions encompassing structural characteristics, herd characteristics, inputs, and outputs. Additionally, the dataset includes LCA results for both HNV farms and key HNV products. The LCA results encompass key environmental indicators: GWP_100_, fossil resource scarcity, land and water use, and biodiversity. The dataset provides a detailed account of all estimations and calculations essential for the comprehensive evaluation of the environmental impacts associated with each farm and HNV product. The excel file contains the following sheets:

1. Assessment_identification
  The assessment identification within the dataset provides a comprehensive description of each farm, encompassing crucial details such as the type of product, country of origin, total agricultural area, and a unique anonymized identification (ID). The geographical context is outlined, including the country, region, location in NUTS 2 and 3 classifications, and precise coordinates. Further metadata includes the date of data collection, the initiation date of the assessment, and information regarding the farm's operational practices—whether conventional or organic. The pedoclimatic characteristics are also documented, encompassing details such as the climate zone, dominant mineral soil type, soil texture, pH levels, annual rainfall, seasonal variations in rainfall during winter and summer, average temperatures, and mean spring temperatures.
2. Herd_description sheet
  Within the 'herd_description' section of the dataset, an account of the farm's livestock is detailed. This includes the specific type of cattle and the diverse breeds produced by the farm. The dataset further provides an exhaustive breakdown of the numbers associated with each animal category, encompassing for beef cattle − suckler cows, milking cows, heifers, calves, steers, bulls −, sheep cattle − breeding ewes, non-productive sheep, rams, lambs− and goat cattle − female goats, strain female young goats, little goats, and billy goats. For each category, the dataset furnishes essential information, including the total number, quantity bought and sold, grazing periods, and the percentage of grazing in both cultivated and semi-natural grasslands. Further details pertaining to feed intake are outlined, incorporating information on grass intake, estimated using E-requirements calculated in the '8. Livestock e-req calculations' sheet and the metabolized energy (ME) for both, semi-natural grasslands and other grasses from different pastures. The dataset further delineates the type, amount, and distinction of purchased or on-farm produced feed and other additional forage. Other aspects such as manure management, the quantity of milk powder utilised, the amount of water consumed, and its source are included.
3. Land use
  This particular sheet addresses the allocation of total agricultural land across various land use types − arable land, arable forage land, and pastures and meadows. This categorisation enables a detailed assessment of the surface area dedicated to forage herbaceous plants. Under the category of pastures and meadows, a further distinction is made among semi-natural grasslands, temporary grasslands and permanent grasslands. Pertinent aspects covered for each field type include the total area, grazed area, yield, and the percentage of legumes. Yield data is derived from national statistics of respective countries for both, pastures and cropland. The “soil amendments” section includes key farming practices such as manure application—distinguishing between organic manure and mineral fertilizer—specifying the corresponding area, quantity, and types also for pesticides, irrigation, and lime. Additional considerations, such as stocking densities for ruminants (both large and small), are also estimated.
4. Other_inputs
  This section covers various aspects of farming operations not covered in preceding sheets, providing an overview of additional critical elements. This includes data on fuel consumption; other organic matter flows, for example the quantities of bedding straw and manure exports; the use of peat and consumption of plastic, specifying the quantities utilised; and other alternative energy sources, such as solar or wood energy.
5. Results_farm_level
  This section shows an exploration of results derived from the environmental impact assessment conducted at the farm level, expressed per hectare, utilising both the Carbon Calculator and Open LCA software. The results encompass greenhouse gas emissions, including CO_2_, CH_4_, and N_2_O, aggregated into a total value in CO_2_ equivalent. Inputs, such as nitrogen (N) from: organic matter, mineral fertilizer usage, manure application, and nitrogen fixation. Outputs, covering organic matter exported, nitrogen in the feed, and associated volatilization. Also, the nitrogen balance values resulted by the difference between N inputs and outputs. Raw estimations of carbon storage per field, distinguishing semi-natural grasslands from other fields. Furthermore, the environmental impact results and average values from the LCA analysis in Open LCA for the following environmental impact categories: GWP100, fossil resource scarcity, land and water use, and biodiversity, derived from the use of the expert system SALCA-BD and expressed as aggregated score numbers per hectare explained in detail along the following sheets “5.1. BD_scores_HNV_farms” and “5.2. SALCA-BD_field_country”. Additional production-related data (yield), presented in various forms including total amount (per kg liveweight or litre) and yield production ratios per hectare, are also detailed.
  5.1. BD_scores_HNV_farms
  This sheet presents the estimates of the impact assessment on biodiversity for each HNV farm. These values are determined by the size of specific land use types, encompassing semi-natural grasslands, permanent grasslands, grain legumes, leys (artificial meadows), and winter cereals. The biodiversity scores are derived from the SALCA-BD expert system method, as outlined in the subsequent sheet. The results showed as a biodiversity score per farm, computed for the entirety of their agricultural area and per hectare, representing the aggregate outcome of the biodiversity scores of the individual land use types.
  5.2. SALCA-BD_field_country
  Biodiversity scores derived from the application of the SALCA-BD expert system [4] per field type (fallow, semi-natural grasslands, permanent grasslands, grain legumes, leys (artificial meadows), and winter cereals) and per country (Greece, France, Finland, Spain and Estonia).
  5.3. Field_operations_example
  This section presents an example of the list of field operations per country and per field type − fallow, semi-natural grasslands, permanent grasslands, grain legumes, leys (artificial meadows), and winter cereals − derived from 41 observations from the farmer´s questionnaires collected. Such list serves as the input for the SALCA-BD expert system, enabling the calculation of biodiversity scores.
6. Results_product_level
  Similar to the “5. Results_farm_level” sheet, this section provides the results at the product level, expressed per kilogram of product. The outcomes stem from the LCA analysis conducted using both the Carbon Calculator and Open LCA software. Total greenhouse gas emissions in CO_2_ equivalent are segregated based on different farming practices, delineating their respective contributions to the overall emissions of the farm. These practices include enteric fermentation, direct and indirect N_2_O emissions from soil, manure management, purchased feed, purchased animals, mineral and organic fertilizers, machinery, fuels manufacturing, farm buildings and materials, and secondary inputs like plastic. The sheet also presents essential parameters such as production yields in terms of tons of liveweight and kg energy corrected milk (ECM), crucial for the detailed calculations. Corrected milk yield values are included alongside the calculated allocation factors between meat and milk. Finally, environmental impacts derived from the LCA analysis by Open LCA, including impact categories such as GWP_100_, fossil resource scarcity, land and water use, and biodiversity, are outlined per product per farm by both functional units, kilograms of product.
  6.1. Peat_emissions_calculations
  This sheet contains estimations of greenhouse gas (GHG) emissions specifically estimated for the utilization of peat. These estimations are limited to farms that explicitly reported the use of peat as bedding. The calculated GHG emissions from peat usage are then incorporated into the final GWP_100_ value corresponding to each respective farm.
7. Results_HNV_food_products
  This section presents the environmental impact results for four categories: fossil resource scarcity, global warming potential, and land and water use of four pivotal High Nature Value (HNV) products − cow milk, goat cheese, beef, and lamb − derived from a LCA analysis based on Agribalyse 3.0 database [5].
  7.1. Feed_substitutions
  This sheet provides a detail presentation of the reference processes selected from Agribalyse 3.0 [5] to derive the environmental impact assessments the four HNV products: beef, lamb, cow milk, and goat cheese. For each product, the primary processes embedded within the respective reference processes are systematically listed. The primary processes belong to each animal category production system, for example: “Calf, conventional, highland milk system, grass fed, at farm gate.” The sheet explores the detailed examination of feed intakes associated with each animal production system, outlining the corresponding substitutions of feed based on the 41 observational inputs from the HNV dataset including the limiting factors employed for these substitutions and the modifications in the feed quantities based on yields. The right side of the sheet offers feed tables that served as sources for these limiting factors and the foundation for all feed substitutions in the analysis.
  7.2. Estimates_nat_french_prod
  This sheet presents estimations of HNV production derived from multiple sources, including the report *“Identification of High Nature Value Farmland in France through Statistical Information and Farm Practices Surveys”* [6]. The percentages of animal production in France are sourced from the national official records and supplemented by data obtained from the dataset encompassing 41 HNV farms. The parameters featured in this sheet focus on beef and dairy cattle and small ruminants providing details for the average ratio, percentages of the total, and production in terms of tons of liveweight per hectare. The estimations encompass the total production in tons of liveweight and the estimated area dedicated to ruminant productions within HNV areas. The estimations included values for cows, heifers, calves, steers, and bulls, small ruminants (sheep and goat).
8. Ruminants E-req calculations
  This sheet outlines the calculations undertaken to derive energy requirements for various animal categories across 41 farms within a one-year production cycle. The list includes big ruminants, including suckler cows, milking cows, heifers, steers (growing bulls), and bulls, as well as small ruminants, encompassing ewes, non-productive ewes, rams, lambs, female goats, little goats, and billy goats. For each category, parameters such as the total number of animals (including those bought and sold), breed information, live weight drawn from literature, and age and yield reported and calculated by the carbon calculator tool, are considered. The sheet further incorporates estimations, including growth rates (g/day), final E-requirements derived through the formulas of the LUKE institute [7], and dressing proportions for beef cattle. These estimations are based on initial values for E-requirement, sourced from the estimated growth rates detailed in tables listed in subsequent sheets labelled “8.1. E-requirements cattle,” “8.3. E-requirements sheep,” and “8.4 E-requirements goats.”
  8.1. E-requirements cattle
  This section provides the formulas and underlying assumptions employed in the assessment of energy requirements for beef cattle based on ruminant energy requirements for meat production for growing heifers and bulls, calves and suckler cows based on the Finish Environmental Institute methodology. Each formula and assumption is systematically presented, with direct references to the respective tables where these estimations are sourced, along with clear indications of the source literature.
  8.2. Dressing percentages
  This section presents the average dressing values extracted from the slaughter data of the year 2017, as reported by the Finnish Food Authority. Dressing percentage is a factor used to calculate carcass weight from a known or estimated liveweight. These values are required for the adjustment of liveweight values influencing the ultimate yield production and, consequently, the energy requirements (E-req) estimation for beef cattle, as outlined in the preceding sheet titled “8. Livestock E-req calculations.”
  8.3. E-requirements sheep
  This section provides the formulas and underlying assumptions employed in the assessment of energy requirements for sheep including the different stages of pregnancy, suckling and maintenance, based on ruminant energy requirements based on the Finish Environmental Institute methodology. Each formula and assumption are systematically presented, with direct references to the respective tables where these estimations are sourced, along with clear indications of the source literature.
  8.4. E-requirements goats
  This section provides the formulas and underlying assumptions employed in the assessment of energy requirements for goats including the different stages of pregnancy, suckling and maintenance, based on mediterranean ruminant energy requirements based on literature. Each formula and assumption are systematically presented, with direct references to the respective tables where these estimations are sourced, along with clear indications of the source literature.

## 4 Experimental Design, Materials and Methods

### 4.1 Study cases and data collection

**Fig. 1:**
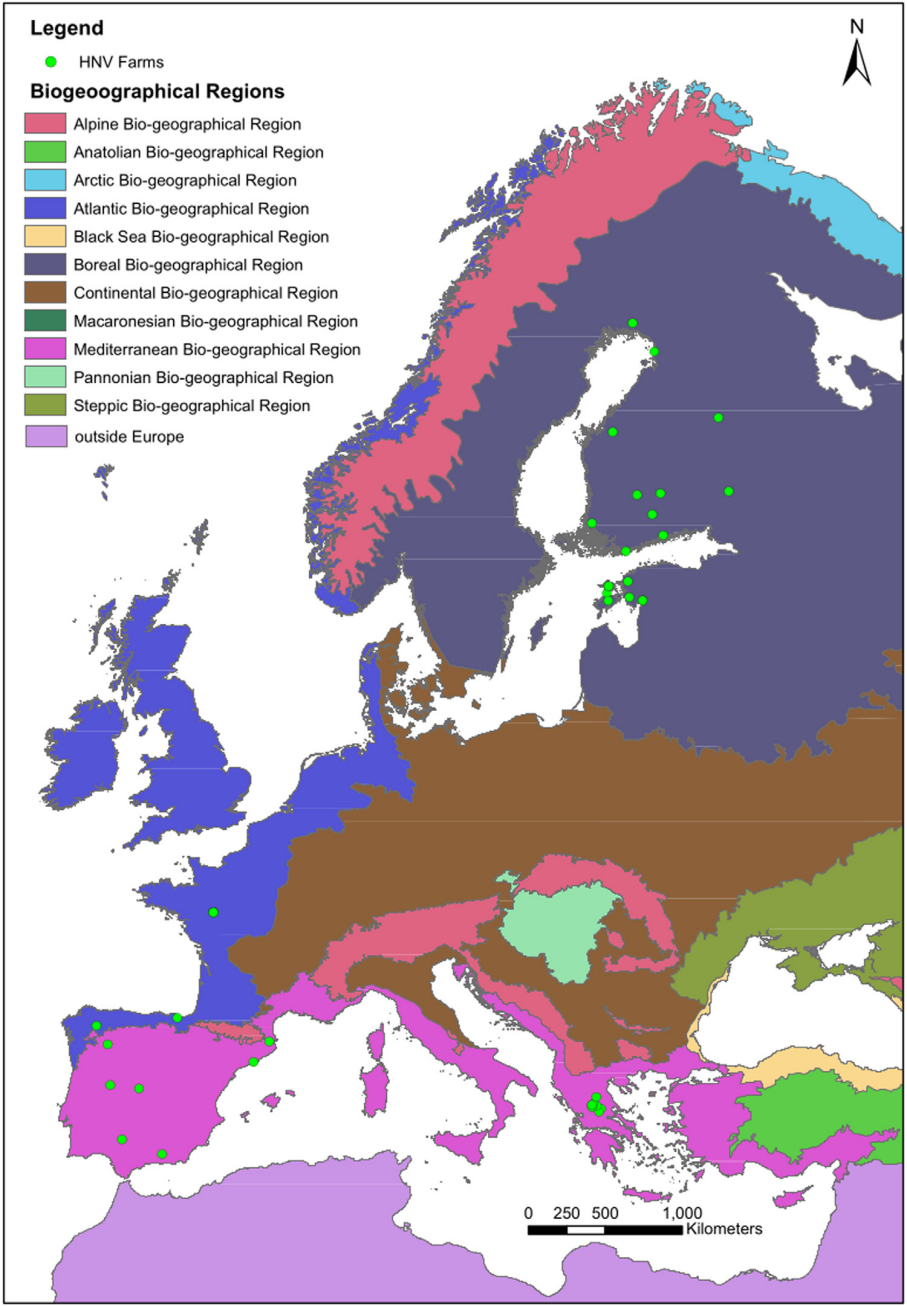
Distribution of the High Nature Value (HNV) farms included in the study within various biogeographical regions in Europe. Base map: European Union, Copernicus Land Monitoring Service 2023, European Environment Agency (EEA).

We established a collaborative network across Greece, Spain, France, Estonia, and Finland to collect comprehensive data on HNV farming systems. This network included key stakeholders such as farmers, researchers, and organisations directly involved with farmers. A total of 41 HNV farms were enrolled in the study, comprising 22 beef cattle, 4 sheep, 2 goats, 5 sheep-goat, 3 dairy, and 5 combined beef-sheep-goat farms. The main inclusion criterion was that ruminant production had to utilise to a large degree semi-natural habitats, that is, “areas of grassland existing as a result of human activity (mowing or livestock grazing), where environmental conditions and the species pool are maintained by natural processes” [8]. Other semi-natural habitats such as coastal pastures in Finland and Estonia and/or forest pastures also in Greece of Spain fulfilled the main criterion. The selection of only ruminants, specifically sheep and cattle, was based on the prevalent agricultural practices within the regions covered in this study, including Northern, Central and Southern Europe. Farmers enrolled in the data collection in different manners. In Finland, our approach involved utilising social media, specifically a Facebook group dedicated to extensive semi-natural grasslands farmers. For international cases, our collaborators facilitated direct engagement with farmers or farmers' organisations. In Spain, we collaborated with “Ganaderas en Red” that facilitated data collection from 13 suitable farms. In Estonia, Greece, and France, we collaborated with researchers directly engaged with farmers in their respective regions with commonly present HNV farming systems. These farms were strategically distributed across key bioregions in Europe, excluding the Continental region (Fig. 1).

Farmers completed a questionnaire covering primary data on farming practices and farm structure (Table 1) (for further details see Tables 1, 2, 3 and 4 of the dataset). National coordinators provided assistance by telephone to address any inconsistencies in the provided data, and help filling some of the data correctly. Primary data covered various aspects of livestock management, including breeds, animal numbers by age groups, grazing intensity, field use, manure management, yield, and other relevant practices on the farms. Critical parameters such as liveweights, growth rates, or forage intakes were modelled based on primary data, literature, and expert assessments.

**Table 1:** Main characteristics of the study region, farm structure, herd structure and inputs of High Nature Value (HNV) farms from Finland, Estonia, Spain, Greece and France (means ± SD).

Biogeographical region	Boreal		Mediterranean		Atlantic
Region	Finland	Estonia	Greece	Spain	France
Mean annual temperature	4.3 °C	6.8 °C	13.9 °C	13.5 °C	12.5 °C
Mean annual precipitation	579 mm	639 mm	642 mm	731 mm	663 mm
Vegetation	Cultivated grassland and cropland: barley, oats, silage, hay.	Cultivated grassland and cropland: barley, oats, hay.	Highlands, mid-valley grasslands, shrubland and forest pastures	Alpine pastures, highlands, mid-valley grasslands, shrubland and open forest pastures	Mid-valley grasslands, permanent pastures.
Typical semi-natural habitat	Coastal and forest pastures	Coastal and forest pastures	Grassland, forest pasture	Grassland, forest pasture	Grassland
Type of production	Beef, sheep, mixed production (beef and sheep)	Beef and dairy	Mixed production (sheep and goat), beef	Sheep, goat, beef, dairy, mixed production (sheep and goat)	Beef and dairy
N farms	11	8	8	9	5
*Farm structure*					
Total on-farm land use (ha)	274 (± 232)	451 (± 414)	30 (± 19)	935 (± 1908)	146 (± 42)
Arable crop land (ha)	22 (± 36)	33 (± 63)	2 (± 4)	0 (± 0)	19 (± 21)
Arable forage land (ha)	113 (± 119)	67 (± 85)	2 (± 4)	1 (± 3)	22 (± 11)
Semi-natural grasslands combined (ha)	138 (± 128)	351 (± 349)	26 (± 19)	934 (± 1910)	105 (± 34)
Communal off-farm land (yes/no)	no	no	Yes	yes	no
Surface of forage herbace (% from total)	93% (± 11)	94% (± 9)	95% (± 9)	100% (± 0)	88% (± 1)
Feed autonomy	90% (± 24)	90% (± 26)	46% (± 32)	83% (± 14)	85% (± 31)
Purchased feeds	Rapeseed, protein crops	Silage, maize, barley	Hay, pea, alfalfa, roughage, straw, maize, barley, soy	Hay, protein crops, silage, straw	Protein crops (cereals), alfalfa, maize
*Herd details*					
Breeds	Eastern finncattle Abeerden angus, Ayrshire holstein, Highland cattle, Charolais, Simmental.	Simmental, Hereford, Limousin, Abeerden angus.	Karagounis-Chiotiko, Greek red.	Charolais, Frisona.	Montbéliardes, Charolais, Limousine.
Herd size	172 (± 195)	204 (± 232)	27 (± 29)	83 (± 91)	202 (± 145)
Suckler cows	66 (± 75)	62 (± 59)	10 (± 14)	34 (± 32)	78 (± 51)
Milking cows	–	11 (± 32)	–	16 (± 33)	10 (± 22)
Heifers	30 (± 35)	50 (± 55)	7 (± 6)	7 (± 7)	42 (± 26)
Steers	26 (± 36)	27 (± 34)	–	–	38 (± 20)
Calves	43 (± 45)	52 (± 52)	9 (± 8)	24 (± 18)	30 (± 24)
Bulls	8 (± 5)	2 (± 2)	1 (± 1)	3 (± 2)	4 (± 1)
Breeds	Finnsheep	–	Crossbred	Castilian sheep, Segurena, Ripollesa, crossbred.	–
Herd size	248 (± 203)	–	265 (± 206)	761 (± 758)	–
Ewes	96 (± 76)	–	132 (± 97)	383 (± 393)	–
Non-reproductive ewes	4 (± 9)	–	–	32 (± 41)	–
Rams	3 (± 4)	–	6 (± 4)	9 (± 8)	–
Lambs	145 (± 115)	–	127 (± 105)	337 (± 317)	–
Breeds	–	–	Skopelou	White goats, crossbred.	–
Herd size	–	–	155 (± 151)	193 (± 110)	–
Goats	–	–	69 (± 65)	98 (± 49)	–
Female goats	–	–	3 (± 6)	16 (± 9)	–
Billy goats	–	–	4 (± 3)	8 (± 8)	–
Goat kids	–	–	80 (± 77)	71 (± 43)	–
*Reproductive management*					
Cattle sold per year	56 (± 92)	72 (± 99)	6 (± 6)	22 (± 27)	68 (± 54)
Sheep and goats sold per year	141 (± 112)	–	157 (± 146)	398 (± 388)	–
Grazing time (% time spent annually) - ruminants*	35% (± 9)	31% (± 14)	71% (± 21)	76% (± 20)	51% (± 19)
Grazing time (% time spent annually) - small ruminants*	47% (± 19)	–	77% (± 25)	70% (± 19)	–
* Potentially available grazing period in each country is limited by the availability of pasture fodder					
Beef live weight of sold animals (t)	28 (± 37)	30 (± 31)	4 (± 3)	8 (± 6)	42 (± 27)
Lamb / goat live weight of sold animals (t)	5 (± 4)	–	3 (± 3)	5 (± 5)	–
Cow milk (l)	–	508 (± 0)	–	250 (± 0)	155 (± 0)
Sheep milk (l)	–	–	24 (± 26)	–	–
Goat milk (l)	–	–	6 (± 6)	4.8 (± 0)	–
*INPUTS*					
*Energy use*					
Diesel used (l)	7873 (± 9907)	17,010 (± 19,024)	1545 (± 1039)	1403 (± 1397)	9525 (± 5900)
*Fertilizers*					
Inorganic fertilizers (number of farms where used)	1 out of 11	1 out of 8	None	none	2 out of 5
Nitrogen (kg/ha)	240 (± 0)	–	–	–	250 (± 141)
Phosphorous (kg/ha)	–	–	–	–	200 (± 0)
Organic nitrogen / manure (N kg/ha)	6 (± 10)	3 (± 8)	1 (± 2)	0 (± 0)	1 (± 2)
*Pesticides*					
Pesticides (number of farms where used)	none	none	1 out of 13	none	1 out of 5
Number of treatments	none	none	1	none	1

**Table 2:** SALCA-BD scores per field type and country.

Production system	Finland	Estonia	France	Greece	Spain
Fallow	15.1	15.1	15.1	15.1	15.1
Grain legumes	5.8	5.6	5.6	5.6	5.8
Leys (artificial meadows)	4.6	5.1	5	4.6	5
Winter cereals	7.2	6.8	7.2	7.2	7.2
Grassland type I (unproductive)	19.5	19.6	19	19.4	19.4
Grassland type II (moderate productive)		11.8	11.8		
Forest pastures				20.4	20.4

### 4.2 Assumptions

We used the best available estimates from a variety of national statistics databases for agricultural yield values. Averaged yields of the main feed crops −such as triticale, alfalfa, barley, faba bean and oat − were determined based on the country-specific production average yields of the last four years in respective regions [7,[9], [10], [11], [12]]. For semi-natural grasslands, we considered yields of 1.8 t DM ha-1 in Finland [13], 2 t DM ha-1 in Estonia [11], 2.2 t DM ha-1 in Greece [14], and 3 t DM ha-1 in France and Spain [12,15]. Semi-natural grasslands in production were included in the total Utilised Agricultural Area (UAA) of each farm as pastures and other field crops. To avoid double counting in the UAA, cover crops were included as a percentage of legumes, with corresponding yields adapted for the field. We assumed 21 % of legumes in semi-natural grasslands in Finland and Estonia [16], 24 % in Spain and Greece [17] and 22 % in France [12]. For cultivated pastures, we assumed 34% in Finland and Estonia [16], and 36 % in Spain [17].

We relied on farmer-reported dietary composition referred to protein feed purchases and other feeds intake. We also included estimated forage intakes during grazing. For this, we applied the same methodology as in [18]) to estimate forage intake originated from semi-natural grasslands and other pastures. We assumed that all crop production was for feed purposes, with no residues left in the field. Calculations were based on specific live weights, ages, growth rates and energy requirements per animal category, breed and metabolisable energy (ME) concentration of low-quality forage. The ME concentrations applied for semi-natural grasslands were 8 MJ kg DM-1 in Finland, 10 MJ kg DM-1 in Estonia [7], 10.2 MJ kg DM-1 in Spain and Greece [15], and 10.56 MJ kg DM-1 in France [19]. For other pastures, the ME concentrations were 11.3 MJ kg DM-1 in Finland, 10.8 MJ kg DM-1 in Estonia [7], 11.3 MJ kg DM-1 in Greece, 11 MJ kg DM-1 in Spain [15] and 11.5 MJ kg DM-1 in France [20].

The energy requirements of cows, calves, growing bulls, and heifers were estimated according to Finnish nutrition requirements [7] (see [18] for additional details). Energy requirements for sheep and goats varied by stage: pregnancy, suckling and maintenance. The nutritional requirements per stage were based on Finnish requirements for sheep [7]. For goat's maintenance stage, an average for free-ranging goat studies was applied [[21], [22], [23]].

We estimated growth rates based on liveweight breed characteristics and age data provided by farmers in the questionnaire for growing bulls, heifers, calves, and lambs. In cases of missing data, we estimated values from relevant literature (e.g., Huuskonen et al., 2017 for Finnish cattle) and use the questionnaire averages for the respective region and production type. We assumed no growth for suckler cows, adult bulls, ewes, and rams. Dressing percentages considered in this study were based on average values from slaughter data from the Finnish Food Authority [7] from a minimum of 44% for dairy cows to a maximum of 55.9 % for a bull >2 year for the beef breed (see Table 8.2. in the repository). To ensure data accuracy, particularly given the major influence of certain parameters (i.e., herd size) on final environmental impact results, we compared production volumes resulting from our calculations with those estimated in the CC (with the functional unit being 1 kg of product).

## 5 Environmental Impact Assessment

### 5.1 HNV farms

We assessed the potential environmental impact of HNV farms through attributional LCA using two types of software: the Solagro Carbon Calculator (CC) [24] and OpenLCA 1.11. The CC tool, tailored to cover EU-27 specifications such as climate, follows international LCA standards (for further details in the methodology, see [24]). The system boundary applied in this study was from cradle to farm gate. We applied the ReCiPe Midpoint 2016 (H) impact method to estimate GWP_100_ (kg CO_2_ eq), FRS (kg oil eq) and LU (m^2^a crop eq), and the AWARE method to assess regionalised WS (m^3^). The functional units used in the LCA were one hectare (ha^−1^) and yield, expressed by kg of product for milk (kg ECM milk^−1^) and meat liveweight (kg LW^−1^). Environmental impact values per product were calculated by dividing impacts per hectare by the total yield of animal products (kg LW and kg ECM milk) hectare^−1^ for each HNV farm. We applied a biophysical allocation method between milk and meat in mixed production systems following the PEF guidance (PEF Guidelines, 2021). Fat and protein content values reported by farmers in the questionnaires were used to estimate the fat and protein corrected milk (FPCM).

Our assessment of the environmental impact was based on a yearly production cycle estimated from 5-year average data reported by farmers. The life cycle inventory based on data collected in the questionnaires, *ad hoc* calculations and results from CC. We included diesel usage in agricultural production (MJ), water consumption (l), land occupation per type of land (ha), mineral fertilisers, feed and plastic purchases based on data collected from the farms. The emissions flows included in the analysis were ammonia (NH_3_), dinitrogen monoxide (N_2_O), methane (CH_4_) from enteric fermentation and other GHG emissions (CO_2_) relevant to manure management, mineral fertilisers, feed and plastic purchases. We used the CC to assess greenhouse gas emissions (CH_4_ and CO_2_, and N_2_O), total N inputs and outputs (N kg ha^−1^) at the farm gate and the contribution of certain farming practices to the overall global warming potential such as manure management or feed purchases for each HNV farm. The emissions resulting from the use of peat as bedding material in 5 out of 41 Finnish and Estonian farms were assessed by using the emission factor of 860 kg CO_2_eq m^3 −1^ and density of 200 kg m^3 -1^ [25]. We excluded from the analysis capital goods, due to minimal machinery and buildings sizes in HNV farming systems.

### 5.2 HNV food products

We estimated the environmental impact of four food products representative of HNV farming systems − beef, lamb, goat cheese, and cow milk (see Table 7 Results food products). We utilised the LCA inventory data sourced from the Agribalyse 3.0 LCA Database [5] using the OpenLCA 1.10.3 software [26]. Agribalyse is a multi-indicator French Life Cycle Inventory (LCI) Analysis database with data for over 2500 products produced in France covering all stages from food production to end use. Agribalyse database has been used for modelling European diets in previous research thus allowing potential standardisations [27]. French data include relative transportation and production differences across Europe by incorporating transportation emissions of products imported from outside of Europe. Further, in the context of our study, France encompasses five bioregions in Europe also used in our HNV dataset. The ruminant production data from Agribalyse underwent assessment by IDELE, the French Institute for Ruminant Production [28]. IDELE´s methodology consists of an ad hoc approach, grounded in French farm data, to categorise the various ruminant production systems in France in different INOSYS case types. Each INOSYS case type corresponds to a production system with standardised values of field sizes and types, feeds, and production outputs among others. We run quantitative and qualitative comparison between the aforementioned LCA inventory in Agribalyse, based on the INOSYS case types, and 41 HNV farms from the HNV dataset to improve the representation of the HNV farming systems in Agribalyse. Therefore, we tailored four LCA processes in Agribalyse to HNV production practices to assess the environmental impact of each HNV food product.

The processes delineated in the LCA inventory Agribalyse 3.1. span from cradle to consumer (end use). Each process commences with an initial process, specifically, the production system of the animal. The initial process defines the foundation for subsequent stages encompassing food preparation and transportation. We focused on the initial processes, this is, the agriculture production process to tailor the HNV product processes. In this study, we defined extensive farming systems as systems that relied on semi-natural grasslands, shrublands or coastal grasslands as the main source of animal feed. We decided on 1.2 livestock unit (LU) as a threshold for extensive ruminant densities on grasslands based on the available processes in the Agribalyse database for this category (class below 1.2 LU), and its use as the limit to receive payments for extensive grazing under the eco-schemes in Europe from 2023 to 2027 [29]. We employed qualitative assessments considering farming practices, feeding strategies and animal output between Agribalyse processes, INOSYS case types and 41 HNV farms to select the most reliable referenced values. We assumed that processing and transportation remained similar to the proxies selected. Thereafter, we selected Agribalyse processes that corresponded to low-input farming practices and in areas with restricted options for intensification such as highlands. The selection had particular importance for cow milk products due to limited cases in the HNV database and for goats, for which data from HNV farms were from conventional farming. Once selection was done and, given the varied inputs and outputs inherent to each LCA process, particularly regarding emissions, we used a conservative approach and made minimal modifications.

We primarily compared farming practices between our dataset and potential Agribalyse production processes such as buildings, types of feed and the use of fertilisers. We substituted feed types according to farming practices common specifically in HNV farming systems (see Table 7.1. Feed substitutions). For example, we substituted grains grown under conventional practices to those grown under organic ones. Similarly, we substituted grasses grown on permanent or cultivated grasslands to forage from grazing. We based the substitutions on the assumption that farmers base the animal dietary composition decisions on limiting factors such as crude protein, NDF, or energy. We verified that substituted feeds will remain similar regarding nutritional values. For this, we utilised the best available estimates on feed nutrition from different French national sources [[30], [31], [32]].

Second, we conducted quantitative calculations based on the original data from 41 farms, relevant reports [33], and national statistics [12] to estimate production output values similar to HNV farming systems (see Table 7.2. Estimates_french_nat_prod). We estimated animal production output per hectare ratios for different animal categories: calves, suckler cows, heifers, steers, growing bulls, lambs and goats. We accounted for the potential extent of HNV farmland in France and particularly, for the extent of grazing livestock according to the following formula:

$$LAI=Extent\;HNVf\;F\times \;\%\;Grazing\;\times \;\frac{Kg\;animal\;output}{Hectares\;HNVf}$$

LAI = Estimated Living Animal Input

Extent HNVf F = Total extent of High Nature Value farmland in France

% Grazing = Total percentage of grazing livestock extent in France kg animal output = litres of milk or kg of liveweight output average per animal category based on 41 HNV farms

Hectares HNVf = average hectares in production per animal category based on 41 HNV farms

The remaining inputs, outputs and emissions, in accordance with HNV farming practices were retained to ensure consistency in the overall environmental impact assessment. We linked the tailored HNV processes to the subsequent LCA processes from different stages, processing, distribution, cooking to complete the scope. The scope used in this part of the study was from cradle to end use. The functional unit used was 100 gr of product. The environmental impact method applied was the ReCiPe Midpoint 2016 (H) to assess the impact on GWP100 (kg CO2 eq ha^−1^) water use (m^3^) and land use (m^2^a crop eq ha^−1^) (see results in SM Table 7. Results_HNV_food_products).

### 5.3 Biodiversity

We estimated the potential biodiversity impact of the farms by using an expert scoring system SALCA-BD [34]. The SALCA-BD scores represent both adverse and beneficial land occupation impacts of agricultural production on terrestrial species diversity at the field scale. The terrestrial species assessed in SALCA-BD (defined as indicator species groups) include grassland and arable flora, birds, small mammals, amphibians, molluscs, spiders, carabid beetles, butterflies, wild bees, and grasshoppers. For each indicator group, the score results from a rating R (1 < R < 5) of the impact of the management option (1, highly damaging to 5, favourable) multiplied by the mean value C (1 < C < 10) of two weighting coefficients. The coefficient C considers the habitat suitability and the relative importance of farming activities (e.g., grazing vs mowing) for the given indicator group in which the management option occurs. Aggregated at farm level, a higher farm score indicates less impact on biodiversity, meaning that the farm has suitable and important fields in terms of habitats for several indicator species groups and uses practices that favour their occurrence [4]. Information on the farming practices applied per country and field type in this study was obtained from questionnaires. We related these practices to the respective practices included in the SALCA-BD method (see example in [1]).

The major field types from SALCA-BD present in the studied HNV farms were fallow, leys (artificial meadows), winter cereals, grain legumes, grassland type I (unproductive), grassland type II (moderately productive) and forest pastures. We matched these with the best matching field types in our dataset: semi-natural grasslands (combination of forest pastures and grassland type I (unproductive) for Greece and Spain, and grassland type I (unproductive) for Finland, Estonia and France), permanent grassland (grassland type II – moderate productive), cultivated grassland (leys – artificial meadows), cereal crops such as oats, barley, maize (winter cereals) and legume and protein crops such as faba beans and peas (grain legumes). The variation among the scores for the same field type in different countries arises from varying management practices typical of the farms in those countries (e.g., mowing frequency) based on the collected questionnaires.

We applied the same method to our four HNV food products. We must account that for a food product, the land use associated is a conjoint of different land use type areas, for instance, to produce cattle, a farmer uses certain hectares of semi-natural grassland and other hectares for cultivated grassland. To account on all the variances regarding land use, we disaggregated the types of land use derived from each LCA process of food product. We estimated an overall biodiversity score by multiplying the land use impact derived from the LCA analysis by the BD score associated to each land use category specified in table 4 by the following formula:

(1) $$OI=\;\sum_{n=1}\left(BDI_{1}\times LO_{1}\right)\;\left(BDI_{2}\times LO_{2}\right)\;.\;.\;.\;.\;.\;.\;\left(BDI_{n}\times LO_{n}\right)$$

Where,

OI: overall impact on biodiversity

BDI: impact on biodiversity per land use type

LO: land occupation value per land use type (m^2^a crop eq ha^−1^)

## Limitations

While this dataset provides novel data from High Nature Value (HNV) farming systems, several limitations influencing the interpretation and generalisation of the results must be acknowledged. The availability of primary data regarding semi-natural grasslands is inherently limited, contributing to challenges in capturing diversity of production of HNV farming systems. Especially data on vegetation composition, productivity or energy and protein content in semi-natural grasslands may differ drastically from those for cultivated and otherwise improved grasslands, due to their predominant location in marginal areas, usually at high altitudes and differences with the surrounded habitats such in coastal grasslands. As a result, we had to make some assumptions and arrive at best estimates in collaborations with experts in other fields such as animal nutrition and related fields were crucial to supplement the data gaps.

The study faced constraints related to timing and travel due to the pandemic, impacting the data collection process. These limitations introduced potential biases, despite concerted efforts to minimise them. The support provided through phone communication aimed to address inquiries promptly, ensuring a robust data collection process.

Therefore, the findings should be considered as indicative rather than universally applicable to all extensive farming systems, even in Europe. The diverse nature of HNV farms across different regions introduces challenges in generalising study findings. Variations in farming practices, climate conditions, and ecological contexts necessitate caution when extrapolating results beyond the specific farms studied. The outcomes offer valuable insights but should be interpreted within the context of the studyʼs geographical and environmental context.

We utilised the Agribalyse dataset to provide improved standardised data for four HNV products. However, such data are based on estimations from various low-input farming systems, some of which may not represent the realities of specifically HNV farming systems. The conservative approach of minimal modifications to Agribalyse data preserved the quantities of feeds and emissions with little modification for the estimates HNV products, which may require a proper ad hoc calculation to enhance their representativeness and reliance, similar to the output values per animal category estimated in the study. Considerable variation in production between the 41 HNV farms may result in potential bias for the selection of the Agribalyse selected processes used as a proxy. The decision of using generalised processes under 1.2 LU limits the representativeness for the HNV farming systems, because on many semi-natural grasslands LU values are in order lower (for example, 0.1–0.6 LU in Ireland [35]). Also, the proxies are representative for France and may not be applicable to all farming systems across Europe. The estimates include only four food products, not covering the whole variety of food products produced by HNV farming systems.

## Ethics Statement

According to The University of Helsinki, there are two committees responsible for reviewing the ethics of non-medical research involving human participants: the Research Ethics Committee in the Humanities and Social and Behavioural Sciences and the Research Ethics Committee of the Faculty of Medicine. These committees have clearly defined instructions on when a committee meeting is required, specifically in the following cases: (1) participation in the research deviates from the principle of informed consent; (2) the research involves intervening in the physical integrity of research participants; (3) the focus of the research is on minors under the age of 15, without separate consent from a parent or carer or without informing a parent or carer in a manner that would allow them to prevent the child's participation in the research; (4) the research exposes participants to exceptionally strong stimuli; (5) the research involves a risk of causing mental harm that exceeds the limits of normal daily life to the research participants or their family members or others closest to them; (6) conducting the research could involve a threat to the safety of participants or their family members or others closest to them. As our research study does not comply with any of these criteria, our study did not require any ethical approval prior to conducting the survey study. We confirm that the authors have read and adhere to the ethical requirements for publication in *Data in Brief*. Further, the current dataset is original, anonymised, and does not involve human subjects, animal experiments, or data collected from social media.

## CRediT Author Statement

**Miriam Torres-Miralles:** conceptualization, methodology, software, validation, formal analysis, investigation, writing, visualization, project administration, funding acquisition. **Philippe Jeanneret:** methodology, software, validation. **Frédéric Joly:** methodology, validation. **Bertrand Dumont:** methodology, validation. **Hanna L. Tuomisto:** methodology, validation, writing – review & editing, supervision. **Irina Herzon:** conceptualization, writing, project administration, supervision.

## Data Availability

FigshareHigh Nature Value farming systems in Europe: a dataset encompassing the environmental impact assessment of farms and extensive ruminant food products. (Original data).
